# Chemical composition and bioactivity of oilseed cake extracts obtained by subcritical and modified subcritical water

**DOI:** 10.1186/s40643-022-00603-6

**Published:** 2022-10-29

**Authors:** Jaroslava Švarc-Gajić, Francisca Rodrigues, Manuela M. Moreira, Cristina Delerue-Matos, Simone Morais, Olena Dorosh, Ana Margarida Silva, Andrea Bassani, Valentin Dzedik, Giorgia Spigno

**Affiliations:** 1 Faculty of Technology, University of Novi Sad, Bulevar Cara Lazara 1, 21000 Novi Sad, Serbia; 2 REQUIMTE-LAQV, Instituto Superior de Engenharia Do Porto, Rua Dr. António Bernardino de Almeida, 431, 4249-015 Porto, Portugal; 3 DiSTAS, Department for Sustainable Food Process, Università Cattolica del Sacro Cuore, Via Emilia Parmense, 84, 29122 Piacenza, Italy; 4 Volgograd State University, 100 Prospect Universitetsky, Volgograd, 400062 Russia

**Keywords:** Oilseed cakes, Subcritical water, Valorization, Phenolics, Sugars, Bioactivity

## Abstract

Recovery of bioactive compounds from biowaste is gaining more and more interest in circular economy models. The oilseed cakes are usually insufficiently exploited by most technologies since they represent valuable matrices abundant in proteins, minerals, and phytochemicals, but their use is mostly limited to feed ingredients, fertilizers or biofuel production. This study was thus focused on the exploration of new valorization pathways of oilseed cakes by subcritical water, representing a safe and economic alternative in the creation of value chains. Pumpkin, hemp, and flax seed cakes were treated with subcritical water in nitrogen and carbon-dioxide atmospheres, as well as in nitrogen atmosphere with the addition of acid catalyst. The degradation of carbohydrate fraction was studied by quantifying sugars and sugar degradation products in the obtained extracts. The extracts obtained under different conditions were further compared chemically with respect to total phenols and flavonoids, as well as to the content of individual phenolic compounds. Furthermore, the effects of subcritical water treatment conditions on antioxidant, antiradical and cytotoxic properties of thus obtained extracts were defined and discussed.

**Graphical Abstract:**

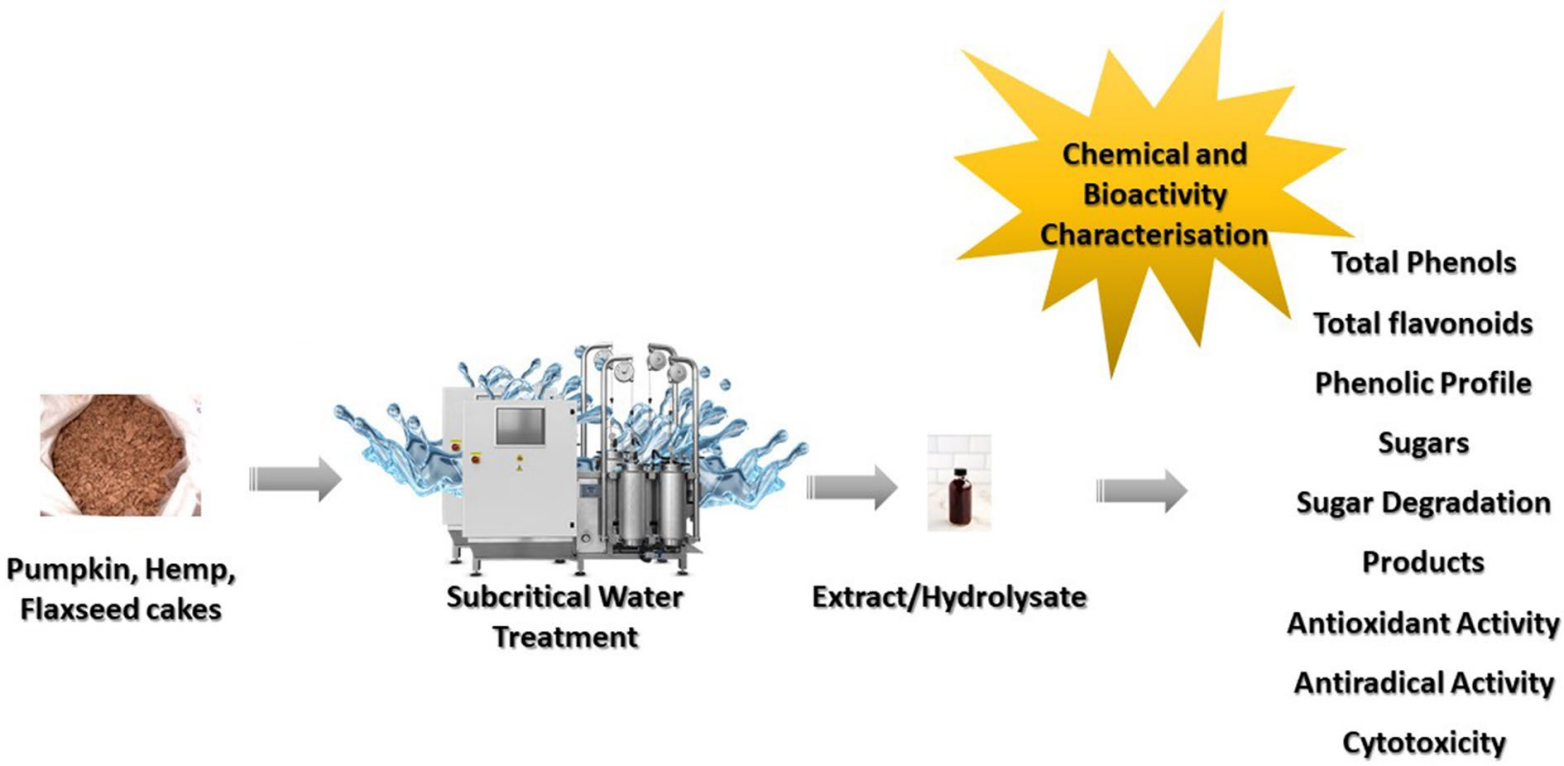

## Introduction

In the last years numerous technologies have been developed for smart waste management in the agri-food sector to increase waste value by converting it into added-value chemicals or energy. New promising approaches relying on different biochemical, thermochemical, and hydrothermal techniques are being more readily implemented into practice. Particularly attractive in this respect is sub-/super-critical water that is used for both extraction and matrix decomposition and its conversion to other chemicals. The popularity of this technique lies in the fact that water is sustainable, safe and cheap solvent whose solvating and reactivity properties can be fine-tuned to target specific chemical classes or processes. Aqueous extracts obtained in such way are free of organic solvents and are potentially compatible with food, cosmetic and pharmaceutical products.

Liquefaction of cellulose from different biomass in subcritical water has often been exploited since cellulose makes great fraction in biomass often remaining underutilized. In subcritical water cellulose is first hydrolysed to oligosaccharides, followed by hydrolysis to glucose. Glucose is further isomerized and dehydrated to 5-HMF and levulinic acid. Özşen (2020) applied a hybrid method of a constant current (up to 2 A) subcritical water treatment to produce levulinic acid from hazelnut shell. Mohan et al. (2015) hydrolysed bamboo biomass with subcritical water applying temperatures ranging from 170 °C to 220 °C targeting maximal yield of reducing sugars (glucose, cellobiose, fructose, xylose and arabinose). The authors found that the yields of reducing sugars increased up to 180 °C, dropping afterwards. The formation of degradation products such as 5-HMF, furfural, aldehydes, dihydroxyacetone, acids, glycol, etc., increased with both reaction time and temperature.

Subcritical water has been reported to be efficient in recovering proteins and amino acids from biomass (Powell et al. 2016; Ramachandraiah et al. 2017), such as rice bran (Sereewatthanawut et al. 2008; Pourali et al. 2009; Xia et al. 2012), yeast (Lamoolphak et al. 2006), and silk (Quitain et al. 2006). Phusunti et al. (2017) extracted proteins from microalgae by subcritical water hydrolysis, converting subsequently the remaining biomass to biofuel. The highest protein recovery (52.68%) was achieved with applying the time of 180 min at 200 °C, whereas highest carbohydrate degradation (51.12%) was achieved at 220 °C for 150 min. The highest lipid content (59.88%) was obtained at 220 °C for 180 min.

Oilseed cakes represent the biomass that remains after oil extraction. Being of favourable chemical and nutritional composition, i.e. rich in proteins, vitamins, minerals, fibres, etc., this biomass is normally used as feed supplement, fertilizer or energy source (Sunil et al. 2015). Alternatively, due to high protein content it can be used for the production of protein isolates and concentrates (Firmanshyah and Yusuf Abduh 2019). Other reported uses of oilseed cakes/meals include their use as a substrate in the production of enzymes, antibiotics, vitamins, pigments, flavours and as source of antioxidants (Ancuta and Sonia 2020).

Valorization of oilseed cakes by subcritical water has not been reported frequently in the literature. Bodoira et al. (2020) used subcritical water/ethanol mixture to extract bioactive compounds from peanut, sesame and pistachio waste. The extracts were characterized in respect to their antifungal properties against *Fusarium verticillioides* which were correlated with the extracted phenolic and lignan compounds. More recently subcritical water extracts of pumpkin, hemp and flax seeds were compared in respect to amino acid, protein, lipid and mineral content (Švarc-Gajić et al. 2020). It was found that the highest relative content of essential amino acids was in pumpkin seed extracts, whereas hemp seed extracts were the richest in flavour amino acids. The authors studied the effects of different gas atmospheres and the effects of acid catalyst on the degree of protein hydrolysis and extractability. This work was thus directed towards the study of carbohydrate fraction hydrolysis from the oilseed biomass in subcritical water, chemical and bioactivity characterization of thus obtained extracts. Pumpkin, hemp and flax seed cakes were extracted by subcritical water in nitrogen and carbon-dioxide atmospheres, as well as in subcritical water with added acid catalyst. Sugars and sugar degradation products were quantified and compared in all obtained extracts. The extracts were further chemically characterized in respect to total phenols and flavonoid contents, as well as detailed phenolics profiles by HPLC analysis. Antioxidant, antiradical and cytotoxic properties of extracts were also evaluated and discussed.

## Experimental

### Samples

Ground oilseed cakes of hemp, pumpkin and flax seed in the form of powder were manufactured by “Beyond” d.o.o. (Belgrade, Serbia). All samples were purchased from a local healthy food retail store from Novi Sad (Serbia).

### Chemicals

Sulfuric acid (Suprapur), hydrochloric acid (Suprapur), calcium carbonate (p.a.) and potassium chloride (p.a.) were purchased from Lachner, Neratovice, Czech Republic. Sodium hydroxide, sodium nitrite, sodium sulfite, sodium chloride and ammonium heptamolybdate were from Centrohem, Stara Pazova, Serbia, all of all pro analysis grade. Potassium sulfate (p.a.) was produced by Alkaloid, Skopje, Macedonia. Copper(II) sulfate (p.a.) and nitric acid (p.a.) were provided by from Zorka Pharma, Šabac, Serbia, whereas n-hexane (≥ 97.0%) was by Panreac Química, SA, Barcelona, Spain. Folin–Ciocalteu reagent, rutin (≥ 94.0%) and gallic acid (≥ 98.0%) were purchased from Sigma-Aldrich (St. Louis, Missouri, USA). Aluminium chloride hexahydrate, sodium carbonate and hydroquinone were provided by Merck (Darmstadt, Germany) and were of p.a. grade. Monopotassium phosphate (p.a.) was purchased of Kemika (Zagreb, Croatia) producer. Phenolphthalein was purchased from Reanal Ltd. (Budapest, Hungary). Ethanol (96%, v/v) was purchased from Sani-Hem d.o.o. (Novi Sad, Serbia).

DPPH radical, TPTZ (2,4,6-Tris(2-pyridyl)-s-triazine, 99%), and 6-hydroxy-2,5,7,8-tetramethylchromane-2-carboxylic acid (Trolox, 98%) were purchased from Sigma-Aldrich (St. Louis, Missouri, USA). Aluminium chloride hexahydrate, sodium carbonate and hydroquinone of p.a. grade were purchased from Merck (Darmstadt, Germany). Iron(III)chloride-6-hydrate (≥ 99%) and ascorbic acid (AA, 99.7%) were obtained from Riedel-de Haën (Seelze, Germany). Methanol and formic acid for HPLC analysis were gradient grade and obtained from Merck. Phenolic compound standards were bought from Sigma-Aldrich (Spain) and their purity was at least above 95%.

Dimethylsulfoxide (DMSO) (99.5%) was purchased from AppliChem (Darmstadt, Germany). Triton X-100 and 3-(4,5-dimethylthiazol-2-yl)-5-(3-carboxymethoxyphenyl)-2-(4-sulfophenyl)-2H-tetrazolium (MTT) were purchased from Sigma Chemical Co. (St. Louis, USA). Dulbecco’s modified Eagle’s medium (DMEM) with GlutaMAX™-I, foetal calf serum, streptomycin, penicillin and amphotericin B were from Invitrogen (Carlsbad, CA, USA).

### Subcritical water treatment

Subcritical water treatment of oilseed cakes was performed in a home-built subcritical water extractor/reactor as described previously (Švarc-Gajić et al. 2017) maintaining sample-to-solvent ratio of 1:30 (w/v) in all extractions. Pressurization of the extraction vessel was performed with 99.999% nitrogen or carbon-dioxide to 20 bars (Messer, Germany). Extraction was performed for 1 h at 160 °C. For each oil seed sample different treatment conditions were tested: ^1^1-SWE: N_2_, 1:30, 160 °C, 20 bar, 1 h; 2-SWE: N_2_, 0.05 mol/l HCl, 1:30, 160 °C, 20 bar, 1 h; 3-CO_2_, 1:30, 160 °C, 20 bar, 1 h, implying nitrogen atmosphere, nitrogen atmosphere with the addition of catalyst (0.05 mol/l HCl), and carbon-dioxide atmosphere without catalyst addition.

The vessel was heated with at approximately 10 °C/min. Agitation was assured at the frequency of vibrational platform of 3 Hz. After extraction, the process vessel was immediately cooled in a flow-through water-bath at 20 ± 2 °C. Depressurization was done by valve opening and purging nitrogen through a valve. Obtained extracts were separated by filtration through Whatman qualitative filter paper, grade 1, and stored in a refrigerator at 4 °C for further analysis, or dried by lyophilization.

### Determination of total phenolic and flavonoids contents

To determine the total phenolic content (TPC) the Folin–Ciocalteu method was used (Markham 1989; Espín et al. 2000). The reaction mixture was prepared by mixing 0.1 ml of the sample, 7.9 ml of distilled water, 0.5 mL of the Folin–Ciocalteu reagent and 1.5 ml of sodium carbonate (20%, w/w). After incubation at room temperature for 1 h, absorbance was measured at 750 nm. The blank was prepared by replacing the samples with distilled water. Triplicate measurements were made for each sample. Gallic acid was used as a calibration standard. The results for TPC were expressed as grammes of gallic acid equivalents per 100 g of sample (g GAE/100 g).

The total flavonoid content (TFC) was measured according to aluminium chloride colorimetric assay based on the procedure described by Markham (1989). Samples (1 ml) were mixed with 5% NaNO_2_ solution (0.3 ml). After 5 min aluminium chloride hexahydrate (10%, 0.3 ml) was added and allowed to stand for further 6 min. Sodium hydroxide (1 mol/dm^3^, 1 ml) was added to the mixture. Immediately, distilled water was properly added to bring the final volume to 10 ml. Blank was prepared using water instead of the samples. The absorbance was measured at 510 nm. Rutin was used as a calibration standard. The results for TFC were expressed as grammes of rutin equivalents per 100 g of sample (g RU/100 g).

### Determination of antioxidant activity

Ferric reducing antioxidant power (FRAP) assay was employed as described in detail by Moreira et al. (2020). FRAP reagent was prepared by mixing acetate buffer (pH 3.6; 300 mM), TPTZ (10 mM in 40 mM HCl solution) and FeCl_3_·6H_2_O (20 mM) in a 10:1:1 ratio. Briefly, 20 μl of extract were mixed with 180 ml of FRAP reagent and the reaction mixture was incubated at 37 °C for 10 min before measure the absorbance spectrophotometrically at 593 nm in 96-wall plates, in a Synergy HT W/TRF Multimode Microplate Reader (BioTek Instruments, Winooski, VT, USA). Standard solutions consisted of AA and the results were expressed as mg AA equivalents per g of dry extract (mg AAE/g dw).

The determination of the DPPH free radical-scavenging assay (DPPH-RSA) was carried out as described by Moreira et al. (2020). Extract (25 μl) was mixed with 200 ml of 0.1 mM DPPH^•^ methanolic solution. The mixture was vortexed and kept in the dark for 30 min before measurement spectrophotometrically at 517 nm. Trolox was used as a calibration standard and the antioxidant activity was expressed as mg Trolox equivalents per g of dry extract (mg TE/g dw).

For ABTS assay, the method of Mendes et al. (2016) was adopted. The ABTS working solution was prepared by mixing equal quantities of 7 mM ABTS solution and 2.45 mM potassium persulfate solution and allowing them to react in the dark for 16 h at room temperature. The solution was then diluted by mixing 5 mL ABTS^•+^ solution with 100 ml water to obtain an absorbance of 0.700 units at 734 nm. Subcritical water extracts (20 ml) were allowed to react with 180 ml of the ABTS^•+^ solution and the absorbance was taken at 734 nm after 6 min. AA was used as standard and the results were expressed as mg AA equivalents per g of dry extract (mg AAE/g dw). For all the assays, triplicate measurements were made for each sample.

### Determination of phenolic compounds profile by HPLC–DAD

Separation and identification of phenolic compounds were carried out by high-performance liquid chromatography (Shimadzu Corporation, Kyoto, Japan) based on the previous described method (Moreira et al. 2020). The HPLC was equipped with diode array detection system, LabSolutions chromatography software and a Gemini C18 reversed phase column (250 × 4.6 mm, 5 μm) from Phenomenex (Alcobendas, Spain) that was kept at 25 °C. A gradient mode using methanol (A) and water (B) with 0.1% formic acid was employed for compounds elution at a flow rate of 1 ml/min. The chromatograms were recorded at 280, 320 and 360 nm. The phenolic compounds identification was carried out by comparing the retention time and UV–VIS spectra with those of pure standards, and their concentration was determined using external standards of the individual phenolic compounds. Lyophilized extracts (50 mg) were dissolved with 1 ml of 20% aqueous methanol, filtered and injected (20 ml). The results were expressed as mg of compound/100 g of dry extract.

### Determination of free reducing sugars and sugar degradation products

The content of free monosaccharides (glucose, xylose, and arabinose) and of acetic acid in the hydrolysis liquors was evaluated by enzymatic kits (Megazyme kit, K-FRUGL, K-Xylose, K-ARGA and K-ACET) and expressed as mg/L of extract. The pH of the liquors was measured directly with a pH-meter (SensION + Ph3, Hach).

The presence and concentration of specific sugar degradation products was evaluated by HPLC with a Perkin Elmer (Norwalk, CT, USA) instrument equipped with a 200 Series pump, a diode array detector (DAD), a Jasco LC-Net II/ADC (Oklahoma City, OK, USA) communication module and operated by ChromNAV Control Center software. A Supelcosil™ LC-18, 250 × 4.6 mm, 5 µm particles fitted column (Supelco) was used with the analysis conditions of: 25 °C, isocratic elution from 0 to 12 min at 0.90 ml/min, linear gradient elution from 12 to 40 min with flow gradient from 0.90 to 1.40 ml/min and then, from 40 to 47 min, with flow gradient from 1.40 to 0.90 ml/min; mobile phase (water:methanol:isopropylalcohol:acetic acid 87:9:2:2). Samples were scanned between 280 and 320 nm and identification and quantification of sugar degradation products (reported as mg/l of extract) was done by using calibration curves defined with external standards: furfural (99%), 5-hydroxymethylfurfural (≥ 99%), 5-methylfurfural (≥ 98%) from Sigma-Aldrich. The liquors were eventually diluted with the mobile phase, filtered (0.45 mm) and injected (20 ml).

### Cytotoxicity assays

Caco-2 clone type C2BBe1 (passage 56–57) and HT29-MTX (passage 41–42) cells have grown in a complete medium, consisting of DMEM supplemented with 10% (v/v) inactivated FBS, 1% (v/v) l-glutamine, 1% (v/v) non-essential amino acids and 1% (v/v) antibiotic–antimitotic mixture (final concentration of 100 U/ml Penicillin and 100 U/ml Streptomycin) at 37 °C and 5% CO_2_ in a water saturated atmosphere in an incubator (CellCulture^®^ CO_2_ Incubator, ESCO GB Ltd., UK). The samples effect on cell viability was measured according to Pinto et al. (2020). The samples effect on cell viability was measured using the 3-(4,5-dimethylthiazol-2-yl)-5-(3-carboxymethoxyphenyl)-2-(4-sulfophenyl)-2H-tetrazolium (MTT) conversion assay. Briefly, cells were seeded separately in 96-well microplates at 25 × 10^3^ cells/well in supplemented DMEM and incubated for 24 h at 37 °C in 5% CO_2_ environment. Extracts, negative control (DMEM), and positive control (1% (v/v) Titron X-100) were added in triplicate to the cell culture. The medium was then changed, and the cells treated with test samples for 24 h. Each treatment was tested in six individual wells. The supernatant was removed, and MTT solution added to each well and incubated for 3 h at 37 °C to allow the formation of formazan crystal. After that the medium was removed, and blue formazan eluted from cells using DMSO. The absorbance was measured at 590 nm with background subtraction at 690 nm. The results were expressed as % of cell viability at different tested extract concentrations.

## Results and discussion

### Total phenols and flavonoids content

Phenolic compounds, known for their great biological potential and wide array of bioactivities (Shakidi and Yeo 2018) were frequently extracted from different matrixes, with both conventional extraction techniques, and modern ones, such as ultrasound or microwave-assisted, that mostly tend to use GRAS (Generally Recognized as Safe) solvents. What distinguishes subcritical water extraction from other techniques is, in the first place the use of a solvent that is environmentally friendly, safe, sustainable, cheap, easily assessable, producing extracts which are totally compatible with food, pharmaceutical and cosmetic products. In addition, solvating properties of subcritical water are tunable, making it possible to target a specific class of phenols, with superior efficiency and remarkable selectivity (Švarc-Gajić 2009).

Total phenols (TPC) and flavonoids (TFC) quantified spectrophotometrically in different oilseed cakes treated under different conditions are presented in Table 1.

**Table 1:** Total phenol and flavonoid contents in oilseed hydrolysates

Sample	TPC (g GAE/100 g)	TFC (g RU/100 g)
Pumpkin		
N_2_	3.26 ± 0.10	0.45 ± 0.00
N_2_, 0.05 M HCl	3.79 ± 0.02	0.36 ± 0.00
CO_2_	3.19 ± 0.01	0.39 ± 0.01
Hemp		
N_2_	3.68 ± 0.04	1.03 ± 0.04
N_2_, 0.05 M HCl	2.78 ± 0.07	0.41 ± 0.00
CO_2_	3.62 ± 0.02	0.58 ± 0.01
Flax		
N_2_	3.56 ± 0.08	0.93 ± 0.01
N_2_, 0.05 M HCl	2.93 ± 0.02	0.54 ± 0.00
CO_2_	3.98 ± 0.04	0.70 ± 0.09

The extraction yield of TPC (Table 1) was comparable for all three oilseed cakes and ranged from 2.78 to 3.98 g GAE/100 g. The influence of water modifiers, both carbon-dioxide and hydrochloric acid, was inconclusive since changes observed in the determined contents under these conditions were not remarkable. In general, in carbon-dioxide atmosphere and with the addition of acidic modifier water reactivity is improved and specific chemical reactions are potentiated, however, this did not seem to affect phenols degradation, which seemed to be relatively stable to concentrations of supplemented acids. However, this reactivity affected total flavonoid content and the greatest yield of flavonoids was observed when extraction was performed in an inert nitrogen atmosphere, without catalyst. Hemp and flax seed cakes showed very close flavonoid yields, ~ 1 g RU/100 g, approximately twofold higher than observed for pumpkin seed cakes. In fact, contrary to what is usually observed in phenols analyses, a low correlation coefficient between the TPC and the TFC, independently on the sample and extraction conditions, was observed (*r* = 0.1942), revealing that different experimental conditions should be applied depending on the sample type, as well as on the family of compounds to be recovered.

Despite of the low correlation between phenolic and flavonoids compounds extracted, subcritical water extraction of phenols and flavonoids from different oilseed cakes seemed to be much more efficient in comparison to other solvents. Teh et al. (2014) compared the efficiency of phenol and flavonoid extraction by methanol, ethanol, hexane, acetone, 80% acetone, 80% methanol and methanol:acetone:water (7:7:6 v/v/v) mixture from hemp, flax and canola cakes. The most efficient showed to be the methanol:acetone:water (7:7:6 v/v/v) mixture which for both phenols and for flavonoids exceeded the efficiency of pure methanol in all three tested samples. For both hemp (733.33 mg GAE/100 g) and flax (774.33 mg GAE/100 g) cakes the contents determined after methanol:acetone:water (7:7:6 v/v/v) extraction were much lower than contents (as extraction yields) determined in this work after subcritical water extraction (Hemp1 3.68 mg GAE/100 g; Flax3 3.98 mg GAE/100 g). Determined maximal contents (as extraction yield) in subcritical water extracts were ~ fivefold higher for both samples in comparison to the highest values determined in methanol:acetone:water extracts. Both total phenols and flavonoids contents determined in this work for subcritical water extracts of pumpkin, hemp and flaxseed cakes were higher in comparison to methanolic extracts of coriander cake (phenols 9.11 mg GAE/g; flavonoids 2 mg CE/g) (Sriti et al. 2019). In fact, subcritical water extracts of oilseed cakes showed to be excellent sources of phenolic compounds and much richer in comparison to sesame cake extract (1.94 mg GAE/g) (Mohdaly et al. 2011), potato peels (2.91 mg GAE/g) (Mohdaly et al. 2010), banana (2.32 mg GAE/g) (Balasundram et al. 2006), carrot (1.52 mg GAE/g) (Kequan and Liangli 2006) and wheat bran (1.0 mg GAE/g) (Kähkönen et al. 1999). Mohdaly et al. (2011) found that the total flavonoid content in sesame cake extract was 0.88 ± 0.02 (mg QE/g) which was ~ 12 fold less than the content in subcritical water extracts Hemp 1.

### Antioxidant activity

**Fig. 1:**
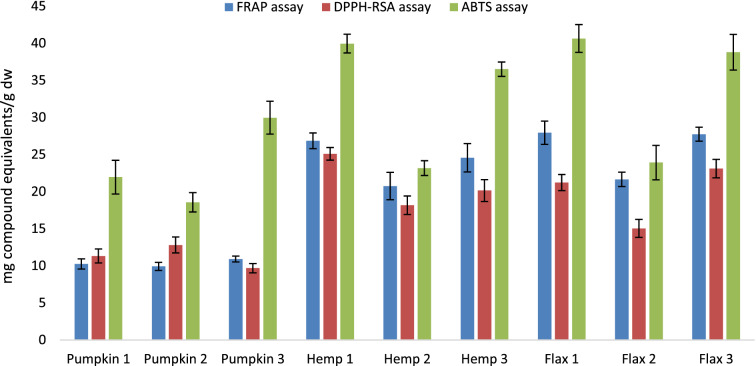
Antioxidant activity of oilseed cakes extracts. Blue bars: ferric reducing antioxidant power (FRAP, results expressed in mg ascorbic acid equivalents/g dry weight); Red bars: 1,1-diphenyl-2-picrylhydrazyl radical scavenging activity (DPPH-RSA, results expressed in mg Trolox equivalents/g dry weight); Green bars: 2,2-azinobis (3- ethylbenzothiazoline-6-sulfonic acid) diammonium salt assay (ABTS, results expressed in mg ascorbic acid equivalents/g dry weight) assays. Results are expressed as mean ± standard deviation (*n* = 3). Subcritical water extraction carried out with a solid to solvent ratio of 1:30 (w/v), 1 h at 160 °C with (1) 20 bar N_2_, (2) 20 bar N_2_ with 0.05 M HCl, (3) 20 bar CO_2_

Several assays are available to estimate the antioxidant activity of plant materials, and each assay has its own specific mechanism to measure these properties. Therefore, for accurate estimation of samples antioxidant activity it is recommended the use of at least two different assays. In the present study, FRAP, DPPH-RSA and ABTS assays were used to evaluate the antioxidant properties of the subcritical water extracts (Fig. 1).

The FRAP values varied from 9.9 ± 0.6 to 28.0 ± 1.6 mg AAE/g dw. The highest reducing power was observed in flax samples, followed by hemp and pumpkin oilseed cakes. As previously observed for phenolic and flavonoid contents, the water modifiers did not improve the reducing power of subcritical water extracts (28.0 ± 1.6 and 27.7 ± 1.0 mg AAE/g dw for conditions 1 and 3 *versus* 21.7 ± 1.0 mg AAE/g dw for condition 2 for flax oilseed cake). Although it was noticed that subcritical water extraction performed in inert nitrogen atmosphere generally resulted in extracts with higher antioxidant activity, the addition of acidic modifier resulted in extracts with the lowest FRAP values. A direct comparison of obtained results with literature cannot be performed as in most studies FRAP values were expressed using Trolox as a standard reference (Nawirska-Olszańska et al. 2013; Deng et al. 2017; Hatica et al. 2018).

DPPH assay of the oilseed cakes extracts had a similar trend to the FRAP assay for the tested extraction conditions. The results (Fig. 1) show that hemp and flax oilseed cakes exhibited the highest antioxidant activity (values ranging from 15.0 ± 1.2 to 25.1 ± 0.8 mg TE/g dw), while pumpkin presented the lowest values (ranging from 9.7 ± 0.6 to 12.8 ± 1.1 mg TE/g dw). Again, extracts with acidic modifier exhibited the lowest antioxidant activity, and the nitrogen atmosphere provided superior values than the carbon-dioxide atmosphere. Our results were lower than the data from flaxseed varieties from China, where values ranged from 32.6 to 46.2 mg TE/100 g (Deng et al. 2017), but higher than the values reported by Izzo et al. (2020) for 27 hemp samples from Italy (6.88 to 19.4 mg TE/g). The differences between our results and the ones reported by previous authors can be explained, not only by the extraction time (30 min) or solvent (methanol) used, but mainly by the extraction technique employed. SWE has been reported as more efficient extraction technique in comparison to conventional extractions enabling to obtain extracts with higher antioxidant activity.

In the ABTS assay, the values ranged from 18.6 ± 1.3 to 40.6 ± 1.9 mg AAE/g dw. Flax 1, hemp 1 and pumpkin 3 showed the highest ABTS radical scavenging activities with the mean of 36.9 mg AAE/g dw, whereas the samples extracted using the acidic modifier (condition 2) revealed the lowest ABTS values with the average of 21.9 mg AAE/g dw. As far as we know, only few studies have employed ABTS assay to determine the antioxidant activity of flax, hemp or pumpkin oilseeds (Nawirska-Olszańska et al. 2013; Deng et al. 2017). However, as previously mentioned for FRAP assay, the referred studies used a different standard to express the obtained results so a direct comparison cannot be performed with the values from the present work.

Overall, results show that antioxidant activity was in accordance with the contents of phenolic and flavonoid compounds, with the oilseed cake extracts obtained employing acidic modifier presenting the lowest values. However, the obtained results in the present study put in evidence that flavonoid compounds are the main contributors to the antioxidant properties of the analysed samples, as a higher correlation was observed with TFC (*r* = 0.6282 for FRAP, *r* = 0.6824 for DPPH• and *r* = 0.7030 for ABTS•) than with TPC (*r* = 0.0741 for FRAP, *r* = 0.2061 for DPPH• and *r* = 0.2720 for ABTS•).

By comparing the antioxidant activity values with HPLC results the opposite behaviour was observed with extracts prepared using condition 2 presenting the highest amount of individual phenolic compounds. Indeed, a low correlation was found between the contents quantified by HPLC and FRAP (*r* = 0.2240), DPPH• (*r* = 0.2235) and ABTS• (*r* = 0.001) values. As previously mentioned, this difference may be explained by increase in extraction efficiency when using HCl modifier, potentiating linkage breaking between cell walls to release phenolic compounds. Furthermore, spectrophotometric assays used for quantification of total phenolic and flavonoid compounds are not selective and specific. During subcritical water treatment of organic matter many neo-formed compounds, due to high water reactivity, increase antioxidant properties of the extracts (Švarc-Gajić et al. 2021).

### Determination of phenolic profile in oil seed extracts

The effects of subcritical water extraction conditions on the phenolic compounds yield from pumpkin, hemp and flax oilseed cakes were investigated by HPLC–DAD. The identification and quantification of the individual phenolic compounds was conducted by comparison of retention time and UV–VIS spectra with those of pure standards (Table 2).

**Table 2:** Individual phenolic compounds identified in oilseed cakes

Compound	Pumpkin1	Pumpkin2	Pumpkin3	Hemp1	Hemp2	Hemp3	Flax1	Flax2	Flax3
Gallic acid	14.9 ± 0.8	26.0 ± 1.3	7.76 ± 0.39	50.9 ± 2.5	99.6 ± 4.9	32.8 ± 1.6	12.2 ± 0.6	103 ± 5	12.7 ± 0.6
Protocatechuic acid	8.35 ± 0.42	10.1 ± 0.5	12.9 ± 0.7	52.4 ± 2.6	20.4 ± 1.0	11.7 ± 0.6	9.51 ± 0.48	57.4 ± 2.9	9.03 ± 0.45
( +)-Catechin	8.96 ± 0.45	8.00 ± 0.40	12.9 ± 0.7	65.2 ± 3.3	58.8 ± 2.9	39.8 ± 1.9	11.4 ± 0.6	13.5 ± 0.7	10.1 ± 0.5
4-Hydroxyphenylacetic acid	14.5 ± 0.7	53.3 ± 2.7	26.1 ± 1.3	21.1 ± 1.0	31.8 ± 1.6	48.3 ± 2.4	34.6 ± 1.7	26.0 ± 1.3	34.6 ± 1.7
4-hydroxybenzoic acid	7.47 ± 0.37	19.9 ± 1.0	9.33 ± 0.47	47.5 ± 2.4	39.8 ± 1.9	9.01 ± 0.45	10.2 ± 0.5	66.6 ± 3.3	10.3 ± 0.5
4-hydroxybenzaldehyde	13.7 ± 0.7	16.3 ± 0.8	9.05 ± 0.45	29.2 ± 1.5	25.3 ± 1.3	16.6 ± 0.8	3.38 ± 0.17	12.4 ± 0.6	10.6 ± 0.5
Chlorogenic acid	11.3 ± 0.6	7.75 ± 0.39	7.10 ± 0.35	9.13 ± 0.46	14.6 ± 0.7	17.8 ± 0.9	11.3 ± 0.6	20.9 ± 1.0	11.3 ± 0.6
Vanillic acid	9.92 ± 0.50	4.71 ± 0.24	10.7 ± 0.5	19.4 ± 0.9	16.3 ± 0.8	17.5 ± 0.9	5.27 ± 0.26	5.67 ± 0.28	5.42 ± 0.27
Caffeic acid	8.07 ± 0.40	4.54 ± 0.23	2.57 ± 0.13	18.4 ± 0.9	16.0 ± 0.6	9.64 ± 0.48	9.66 ± 0.48	12.0 ± 0.6	9.41 ± 0.47
Syringic acid	3.34 ± 0.17	3.16 ± 0.16	3.56 ± 0.18	54.8 ± 2.7	51.8 ± 2.6	37.0 ± 1.8	15.9 ± 0.8	14.3 ± 0.7	15.5 ± 0.8
(-)-Epicatechin	4.14 ± 0.21	11.83 ± 0.59	3.93 ± 0.20	18.8 ± 0.9	10.1 ± 0.5	14.5 ± 0.7	29.9 ± 1.5	27.9 ± 1.4	33.2 ± 1.7
*p*-Coumaric acid	2.77 ± 0.14	2.62 ± 0.13	3.89 ± 0.19	8.37 ± 0.42	6.79 ± 0.34	7.57 ± 0.38	2.16 ± 0.11	4.75 ± 0.24	2.78 ± 0.14
Ferulic acid	2.26 ± 0.11	8.91 ± 0.45	7.58 ± 0.38	13.1 ± 0.6	13.9 ± 0.7	6.72 ± 0.34	3.38 ± 0.17	8.72 ± 0.44	5.32 ± 0.27
Sinapic acid	2.32 ± 0.12	5.10 ± 0.25	5.65 ± 0.28	6.07 ± 0.30	10.4 ± 0.5	3.50 ± 0.18	7.86 ± 0.39	9.68 ± 0.48	3.71 ± 0.19
Naringin	6.47 ± 0.32	1.27 ± 0.06	5.90 ± 0.29	18.1 ± 0.9	29.9 ± 1.5	28.6 ± 1.4	9.28 ± 0.46	6.39 ± 0.32	8.58 ± 0.43
Rutin	6.14 ± 0.31	6.61 ± 0.33	4.62 ± 0.23	12.2 ± 0.6	44.9 ± 2.3	12.0 ± 0.6	4.43 ± 0.22	5.00 ± 0.25	4.54 ± 0.23
Resveratrol	9.18 ± 0.46	1.98 ± 0.10	9.33 ± 0.47	1.33 ± 0.07	1.81 ± 0.09	1.89 ± 0.09	2.23 ± 0.11	1.61 ± 0.08	1.99 ± 0.10
Quercetin-3-*O*-glucopyranoside	17.82 ± 0.89	4.08 ± 0.20	6.91 ± 0.35	4.16 ± 0.21	4.38 ± 0.22	7.08 ± 0.35	4.84 ± 0.24	7.69 ± 0.38	5.07 ± 0.25
Phloridzin	5.33 ± 0.27	1.71 ± 0.09	3.75 ± 0.19	2.13 ± 0.11	1.23 ± 0.06	1.70 ± 0.08	7.75 ± 0.39	1.91 ± 0.10	7.00 ± 0.35
Cinnamic acid	3.72 ± 0.19	2.61 ± 0.13	2.72 ± 0.14	1.94 ± 0.10	2.20 ± 0.11	2.46 ± 0.12	2.31 ± 0.12	6.29 ± 0.31	2.19 ± 0.11
Myricetin	28.6 ± 1.4	31.5 ± 1.6	28.1 ± 1.4	39.9 ± 2.0	49.9 ± 2.5	57.2 ± 2.9	32.1 ± 1.6	32.1 ± 1.6	32.1 ± 1.6
Kaempferol-3-*O*-glucoside	9.09 ± 0.45	7.47 ± 0.37	8.73 ± 0.44	7.83 ± 0.39	6.80 ± 0.34	8.66 ± 0.43	14.2 ± 0.7	7.17 ± 0.36	13.3 ± 0.7
Kaempferol-3*-O*-rutinoside	9.21 ± 0.46	6.02 ± 0.30	6.07 ± 0.30	5.80 ± 0.29	4.76 ± 0.24	8.79 ± 0.44	5.82 ± 0.29	7.26 ± 0.36	8.26 ± 0.41
Naringenin	3.32 ± 0.17	1.18 ± 0.06	3.44 ± 0.17	3.70 ± 0.19	4.08 ± 0.20	3.61 ± 0.18	8.41 ± 0.42	2.64 ± 0.13	4.97 ± 0.25
Quercetin	7.93 ± 0.40	10.0 ± 0.5	8.02 ± 0.40	6.17 ± 0.31	7.88 ± 0.39	9.02 ± 0.45	12.4 ± 0.6	10.3 ± 0.5	11.4 ± 0.6
Phloretin	1.19 ± 0.06	2.20 ± 0.11	1.25 ± 0.06	0.77 ± 0.04	3.22 ± 0.16	1.75 ± 0.09	1.24 ± 0.06	7.87 ± 0.39	1.00 ± 0.05
Tiliroside	3.21 ± 0.16	3.81 ± 0.19	3.25 ± 0.16	2.67 ± 0.13	4.27 ± 0.21	3.34 ± 0.17	4.41 ± 0.22	4.50 ± 0.22	4.50 ± 0.22
Kaempferol	8.97 ± 0.45	9.23 ± 0.46	8.98 ± 0.45	6.87 ± 0.34	8.10 ± 0.40	8.72 ± 0.44	8.70 ± 0.44	8.51 ± 0.43	8.82 ± 0.44
Pinocembrin	4.14 ± 0.21	4.12 ± 0.21	4.22 ± 0.21	3.42 ± 0.17	4.31 ± 0.22	5.81 ± 0.29	4.04 ± 0.20	4.11 ± 0.21	4.10 ± 0.20
All phenolic compounds	236 ± 6	276 ± 11	228 ± 6	532 ± 19	593 ± 23	433 ± 14	289 ± 9	497 ± 23	292 ± 9

Results are expressed as mean ± SD (mg of compound per 100 g dw, *n* = 3)

Subcritical water extraction carried out with a solid to solvent ratio of 1:30 (w/v), 1 h at 160 °C with (1) 20 bar N_2_, (2) 20 bar N_2_ with 0.05 M HCl, (3) 20 bar CO_2_

A total of 29 different polyphenolic compounds were identified in the extracts from oilseed cake samples of pumpkin, hemp and flax. In accordance with TPC and TFC data, hemp and flax oilseed extracts presented the highest amount of all phenolic compounds identified and quantified by HPLC in comparison to pumpkin samples. However, a low correlation was found between the contents determined by HPLC and TPC (*r* = 0.1714) and TFC (*r* = 0.0431) values. Further, it was observed that extracts obtained employing the extraction condition 2 (using the acidic modifier) resulted in higher levels of phenolic compounds. This could be related to facilitated breakage of some polyphenol cell walls linkages increasing the total amount of quantified compounds, but affecting to different extent individual phenolic compounds.

Regarding the compounds belonging to the flavonoid family, the flavonol myricetin was detected in all analysed samples for all tested conditions with values ranging from 28.1 ± 1.4 to 57.2 ± 2.9 mg/100 dw for pumpkin 3 and hemp 3, respectively. ( +)-Catechin was also quantified in all samples, but its amount was at least threefold higher in hemp extracts (65.2 ± 3.3, 58.8 ± 2.9 and 39.8 ± 1.9 mg/100 dw for hemp 1,2 and 3, respectively) than in the pumpkin or flax oilseed cakes (values ranging from 8.00 ± 0.40 to 13.5 ± 0.7 mg/100 dw). In the case of (−)-epicatechin, the highest level was found in flax (27.9 ± 1.4 to 33.2 ± 1.6 mg/100 dw), followed by hemp (10.1 ± 0.4 to 18.8 ± 0.9 mg/100 dw) and pumpkin (3.93 ± 0.20 to 11.8 ± 0.6 mg/100 dw). Rutin and naringin were also found in at least twofold higher amount in hemp extracts, than in flax or pumpkin oilseeds extracts, with the highest value being reported for the extract obtained at condition 2 (29.9 ± 1.5 and 44.9 ± 2.2 mg/100 dw for naringin and rutin, respectively). Similar flavonoid profile was reported by other authors for these types of samples; however, the main contributors to the total amount of phenolic compounds differed from the samples in study. Izzo et al. (2020) studied hemp samples and reported that quercetin-3-glucoside (28.59 mg/100 g) was the major contributor to the phenolic composition. For pumpkin sample, Kulczyński and Gramza-Michałowska (2019) reported the highest values for rutin (46.9 ± 0.1 mg/100 dw) and kaempferol (36.24 ± 0.08 mg/100 dw), while in the present study myricetin was extracted in higher levels.

In the case of compounds from phenolic acid family, gallic acid was one of the major contributors to the total amount of phenolic compounds quantified in oilseed cakes, with the highest values reported for the samples extracted using the acidic modifier (condition 2). The amount of syringic acid in hemp (37.0 ± 1.8 to 54.8 ± 2.7 mg/100 dw) and flax (14.3 ± 0.7 to 15.9 ± 0.8 mg/100 dw) in comparison to pumpkin (3.16 ± 0.16 to 3.56 ± 0.18 mg/100 dw) oilseed samples should also be highlighted in comparison to other phenolic acids detected. In addition, 4-hydroxyphenylacetic and 4-hydroxybenzoic acids and 4-hydroxybenzaldehyde were also some of the main contributors for the total amount of phenolic compounds for the studied samples. Our results for phenolic acids composition in flax and hemp extracts differed from the data obtained by other researchers, who reported that *p*-coumaric and ferulic acids were the predominant compounds in flaxseed sample from Canada (Alu’datt et al. 2013) and hemp samples from Italy (Izzo et al. 2020) obtained after conventional extraction with methanol. In another study (Deng et al. 2017), syringic and 4-hydroxybenzoic acids were also the most abundant phenolic acids in flax samples, with values ranging from 1.37 to 18.3 mg/100 g and from 0.24 to 6.37 mg/100 g, respectively. Regarding the phenolic acids profile of extracts from pumpkin samples, Kulczyński and Gramza-Michałowska (2019) found a similar composition; however, protocatechuic and caffeic acids were the major contributors (values ranging from 4.50 ± 0.11 to 52.55 ± 0.04 and 8.96 ± 0.07 to 118.83 ± 0.58 mg/100 g dw, respectively).

Previously described differences in the contents of individual phenolic compounds from our study in comparison to literature values can be partly attributed to the phenolic acid profiles of the specific varieties and locations. In addition, the different extraction solvents, and extraction techniques (conventional extractions *versus* SWE) also affected the extraction effectiveness of the phenolic compounds in oilseed cakes.

Despite literature reports that antioxidant properties of extracts are due to synergetic effects of all extracted compounds, as the amount of identified and quantified phenolic compounds was lower than 10 mg/100 g dw, it is estimated that they were not the major contributors in the antioxidant properties exhibited by hemp, flax, and pumpkin oilseed cakes.

### Determination of reducing sugars and sugar degradation products

Biomass hydrolysates obtained by subcritical water are usually quite acidic due to the formation of various organic acids in the course of matrix decomposition. The acidity and the content of the most abundant cellulose and hemicellulose degradation products, such as acetic acid and reducing sugars, were measured in all oilseed hydrolysates (Table 3).

**Table 3:** The contents of acetic acid and reducing sugars in oilseed cakes hydrolysates

Sample	Treatment	pH	mg Glu/l	mg Xyl/l	mg Arab/l	mg Acet/l
Pumpkin	N_2_	5.81 ± 0.01	45.71 ± 1.10	6.72 ± 0.21	19.99 ± 1.02	743.67 ± 14.37
	N_2_, 0.05 M HCl	3.50 ± 0.00	105.28 ± 3.98	0.64 ± 0.19	24.51 ± 1.5	463.14 ± 5.58
	CO_2_	5.68 ± 0.01	28.53 ± 1.10	1.56 ± 0.10	16.95 ± 0.91	375.69 ± 3.06
Flax	N_2_	4.95 ± 0.01	37.88 ± 1.21	31.91 ± 0.71	84.37 ± 0.79	284.22 ± 1.89
	N_2_, 0.05 M HCl	3.25 ± 0.00	148.4 ± 8.11	6.65 ± 0.22	41.7 ± 0.68	64.72 ± 3.06
	CO_2_	5.11 ± 0.01	39.21 ± 0.63	32.83 ± 0.23	336.33 ± 2.71	352.37 ± 2.28
Hemp	N_2_	4.80 ± 0.01	5.57 ± 0.55	20.45 ± 1.10	8.36 ± 0.62	358.63 ± 2.28
	N_2_, 0.05 M HCl	3.21 ± 0.01	255.54 ± 3.15	76.14 ± 2.2	88.52 ± 1.58	336.57 ± 6.83
	CO_2_	5.15 ± 0.01	10.61 ± 0.82	27.81 ± 1.2	24.45 ± 0.71	353.73 ± 3.37

Data represent mean values (n = 3) ± SD

*Glu* glucose; *Xyl* xylose; *Arab* arabinose; *Acet* acetic acid

As expected, the presence of the HCl catalyst gave more acidic extracts. Despite the formation of carbonic acid, by CO_2_ dissolution, the acidity of hydrolysates obtained in nitrogen and carbon-dioxide atmospheres, was close, probably because formed carbonic acid was rapidly consumed in degradation reactions. All samples of oilseed cake hydrolysates were strongly coloured, due to pronounced reactions in organic matter, compromising the reliability of enzymatic assays that were used for sugar measurement and relying on spectrophotometric measurement (Table 3). In general, it was observed that cellulose hydrolysis with acid catalyst led to the highest glucose concentrations, being several folds higher in comparison to other treatment conditions for all oilseed cake samples. In line with the previous results hemp and flax hydrolysates presented the highest amounts of glucose (255.54 and 148.4 mg/l, respectively). A higher correlation between glucose amounts and FRAP (*r* = 0.6607), DPPH (*r* = 0.3631) and ABTS (*r* = 0.2805) was found than the one reported for HPLC results. These results demonstrate that glucose constituents can be the main compounds responsible for the extracts bioactivities. Xylose, originating from hemicellulose fraction, in moderate concentration was determined in flax and hemp seed samples, whereas in pumpkin seed cake hydrolysates it was very low. Arabinose content, originating from hemicellulose as well, appeared not to be correlated with hydrolysis conditions and was the highest in the flax cake samples, especially in hydrolysates obtained in carbon-dioxide atmosphere (336.33 mg/l). In practically all samples acetic acid formation was quite pronounced, contributing to the acidity of hydrolysates. Acetic acid results showed no specific correlation with both the treatment conditions and the oilseed cake, probably because this organic acid can be formed from different parent compounds through different pathways. The highest content, however, was registered in the pumpkin seed hydrolysate (743.67 mg/l), whereas in flax seed hydrolysate obtained with acid catalyst, its content was more than 11-fold lower (64.72 mg/l).

The reactivity of water causes the formation of different compounds that are not originally present in the sample (Švarc-Gajić et al. 2021). The major sugar degradation products, namely furfural, 5-hydroxymethylfurfural (5-HM) and 5-methyl-furfural (5-MF) were quantified in all hydrolysates (Table 4). These products are often considered limiting factors for the safety of subcritical water extracts/hydrolysates, thus their quantification is important and relevant.

**Table 4:** The content of sugar degradation products in oilseed cake hydrolysates

Sample	Treatment	5-HMF (mg/l)	Furfural (mg/l)	5-MF (mg/l)
Pumpkin	N_2_	< 1	< 1	< 1
	N_2_, 0.05 M HCl	67.44 ± 1.72	37.94 ± 0.21	11.10 ± 0.02
	CO2	< 1	< 1	< 1
Flax	N_2_	< 1	1.45 ± 0.02	< 1
	N_2_, 0.05 M HCl	112.44 ± 10.11	137.90 ± 1.41	13.84 ± 0.48
	CO_2_	3.33 ± 0.07	13.60 ± 0.17	11.17 ± 0.20
Hemp	N_2_	< 1	< 1	< 1
	N_2_, 0.05 M HCl	74.03 ± 13.62	79.09 ± 5.88	12.31 ± 0.24
	CO_2_	< 1	< 1	< 1

Data represent the means of replicates (*n* = 3) ± SD

*5-HMF* 5-hydroxymethylfurfural; *5-MF* methyl-furfural

The results of sugar degradation products show higher values for the acid-catalysed hydrolysates. This can be explained by the stronger operating conditions due to the presence of HCl that promotes not only the degradation of cellulose and hemicellulose to glucose and xylose, respectively (as mentioned above), but also the subsequent degradation of glucose into 5-HMF and of xylose into furfural (Bassani et al. 2020).

The highest contents of 5-HMF and furfural were quantified in flax hydrolysates, being 112.44 mg/l and 137.90 mg/l, respectively. The values were lower, though, than those found in liquors from acid hydrolysis of wheat straw (Vadivel et al. 2017) carried out at 121 °C, 45 min with 4.7% sulphuric acid. The quantified contents of 5-MF were close in all hydrolysates (11.10–13.84 mg/l).

### Cytotoxicity

The viability of the two malignant intestinal cell lines, Caco-2 and HT29-MTX, were evaluated after exposure during 24 h to the different samples at 3 distinct concentrations (250, 500 and 1000 µg/ml). Tables 5 and 6 summarize the obtained results.

**Table 5:** The effects of different extracts exposure on the Caco-2 cells cell viability

Sample	Control	Cell viability (%)		
		250 µg/mL	500 µg/mL	1000 µg/mL
Pumpkin 1	100.00 ± 9.41	97.17 ± 13.63^b^	119.95 ± 21.12^a^	115.95 ± 12.13^a,1^
Pumpkin 2		96.02 ± 18.26^a^	97.13 ± 16.46 ^a^	61.93 ± 9.77^b,2^
Pumpkin 3		105.20 ± 13.38	114.75 ± 16.06	118.32 ± 19.59^1^
Hemp 1		58.11 ± 8.02	67.43 ± 9.92	61.05 ± 11.05^1^
Hemp 2		74.85 ± 13.53^a^	67.29 ± 12.37^a^	8.93 ± 1.17^b,2^
Hemp 3		82.21 ± 10.48	76.77 ± 15.42	92.19 ± 15.42^1^
Flax 1		86.07 ± 16.76^1,2^	76.40 ± 10.10^2^	88.72 ± 18.13^1^
Flax 2		85.18 ± 16.96^1^	94.98 ± 19.77^1^	96.10 ± 18.82^1^
Flax 3		72.89 ± 10.67^a,2^	55.90 ± 7.88^b,3^	47.61 ± 10.30^b,2^

Values are expressed as mean ± standard deviation (*n* = 6)

Different letters (a, b, c) in the same row indicate significant differences between concentrations of the same sample extracted by different methodologies (*p* < 0.05), according to Tukey’s HSD test

Different superscript numbers (1,2,3) in the same species at the same concentration represent significant differences (*p* < 0.05) between the extraction technique employed (*p* < 0.05), according to Tukey’s HSD test

Subcritical water extraction carried out with a solid to solvent ratio of 1:30 (w/v), 1 h at 160 °C with (1) 20 bar N_2_, (2) 20 bar N_2_ with 0.05 M HCl, (3) 20 bar CO_2_

**Table 6:** The effects of different extracts exposure on the viability of HT29-MTX cells

Sample	Control	Cell viability (%)		
		250 µg/mL	500 µg/mL	1000 µg/mL
Pumpkin 1	99.99 ± 14.42	99.46 ± 9.76^b^	116.49 ± 6.72^a^	113.90 ± 9.46^a,1^
Pumpkin 2		89.98 ± 9.62	80.89 ± 12.54	77.38 ± 7.85^2^
Pumpkin 3		87.87 ± 8.15	84.92 ± 10.30	84.48 ± 7.68^2^
Hemp 1		93.09 ± 11.24	88.96 ± 10.90	82.75 ± 10.55
Hemp 2		98.64 ± 11.46^a^	101.32 ± 9.61^a^	81.90 ± 5.00^b^
Hemp 3		84.00 ± 5.60^b^	85.73 ± 1.44^b^	103.70 ± 4.77^a^
Flax 1		87.25 ± 13.54^2^	84.19 ± 15.18	78.45 ± 12.30
Flax 2		77.48 ± 12.84^2^	78.19 ± 11.63	76.53 ± 9.95
Flax 3		100.79 ± 6.32^a,1^	84.47 ± 5.24^b^	70.55 ± 4.70^c^

Values are expressed as mean ± standard deviation (*n* = 6)

Different letters (a, b, c) in the same row indicate significant differences between concentrations of the same sample extracted by different methodologies (*p* < 0.05), according to Tukey’s HSD test

Different superscript numbers (1,2,3) in the same species at the same concentration represent significant differences (*p* < 0.05) between the extraction technique employed (*p* < 0.05), according to Tukey’s HSD test. Subcritical water extraction carried out with a solid to solvent ratio of 1:30 (w/v), 1 h at 160 °C with (1) 20 bar N_2_, (2) 20 bar N_2_ with 0.05 M HCl, (3) 20 bar CO_2_

Caco-2 is a human intestinal cell line isolated from colon adenocarcinoma that forms polarized monolayers, achieving a differentiation similar to enterocytes. HT29-MTX was obtained from HT-29 cells and have the ability to secret small amounts of mucins, important compounds for absorption process. Both cell lines are usually employed to study the intestinal effects of different matrixes (Pinto et al. 2020, 2021; Lameirao et al. 2020).

Regarding Caco-2 cells (Table 5), it is possible to observe that pumpkin 1 and 3 did not decrease cell viability at different concentrations, with values ranging between 97.17 ± 13.63% and 118.32 ± 19.59%. Oppositely, the pumpkin 2 (extracted with N2, 0.05 mol/l HCl, 1:30, 160 °C, 20 bar, 1 h) resulted in viability of 61.93 ± 9.77% at the highest concentration tested, showing a considerable degree of cytotoxicity. In what concerns to hemp, sample 3 affected to highest extent Caco-2 viability. Once again, the sample extracted under the second condition (N_2_, 0.05 mol/l HCl, 1:30, 160 °C, 20 bar, 1 h) led to lowest cell viability at the highest concentration tested (8.93 ± 1.17%).

The extraction of flax under conditions 1 (N_2_, 1:30, 160 °C, 20 bar, 1 h) and 2 (N_2_, 0.05 mol/l HCl, 1:30, 160 °C, 20 bar, 1 h) led to similar results (ranging between 85.18 ± 16.96% and 96.10 ± 18.82%), while the extraction under condition 3 (CO_2_, 1:30, 160 °C, 20 bar, 1 h) caused the lowest cell viability (47.61 ± 10.30%). These effects were probably associated with the bioactive compounds extracted, as previously reported, particularly when HCl was used in the extraction (condition 2). By using acidic modifier, the linkage between cell walls and bioactive compounds was more easily broken, releasing cytotoxic compounds. In addition, increased reactivity of subcritical water with the modifier may have led to formation of cytotoxic compounds that affected cytotoxicity studies. Neoformation of cytotoxic compounds in subcritical water should not be neglected taking into consideration tremendous reactivity of such medium and numerous chemical reactions that sample molecules undergo.

Table 6 summarizes the results obtained for HT29-MTX cells. As it possible to observe, the results were similar for pumpkin, hemp and flax, without huge differences between the extraction conditions. The viabilities achieved for all tested concentrations were always above 75%, not being classified as cytotoxic. In some samples, such as flax seed extracted under condition 3 (CO_2_, 1:30, 160 °C, 20 bar, 1 h) or pumpkin seed extracted under condition 2 (N_2_, 0.05 mol/l HCl, 1:30, 160 °C, 20 bar, 1 h), a significant decrease of viability after exposure to the highest concentration was observed. However, comparing these results with the ones obtained for Caco-2, the effects were less severe.

To the best of our knowledge, this is the first study that evaluated the intestinal effects of flax and hemp oilseed cakes extracts. Nevertheless, Medjakovic et al. (2016) already screened the effects of hydro-ethanolic extract of pumpkin seeds (100–500 µg/ml) from the Styrian pumpkin (*Cucurbita pepo* L. subsp. pepo var. styriaca) on the viability of human epithelial colorectal adenocarcinoma (Caco-2). According to the authors, the crude pumpkin seed extract inhibited the cell growth, achieving similar results to the present study. As an overall conclusion, the Caco-2 samples were more sensible to the tested extracts, with condition 2 leading to more significant decrease of cell viability. This was already observed by our research group using other matrixes. These results are also in line with the phenolic profiles previously described and discussed relating this bioactivity with phenolic compounds.

In addition to proven bioactivities of the oilseed cakes extracts obtained in this work by subcritical water, such as cytotoxic, antioxidant and antiradical, the extracts were high in valuable compounds, such as different phenolic acids, flavonoids, glucose, arabinose and acetic acid. Rich phenolic profile seen in obtained extracts, non-toxicity of used solvent, as well as aqueous nature, gives a perspective to these extracts to be used in food, cosmetic and pharmaceutical industries. Produced sugars and organic acids, on the other hand, can further be used by chemical industry for conversion to other molecules, or be used for biofuel production.

## Conclusions

Pumpkin, hemp, and flax seed cakes were treated with subcritical water and modified subcritical water for simultaneous extraction and degradation of matrix constituents. The extracts/hydrolysates were characterized chemically and biologically. The content of total phenols and flavonoids, as well as the full chemical profile of phenolic compounds, were defined in the extracts. In addition, the content of reducing sugars and sugar degradation products was calculated. The bioactivity of extracts/hydrolysates was evaluated in respect to antioxidant/antiradical properties and cytotoxicity.

The extraction yield of total phenols was comparable for all three oilseed cakes and ranged from 2.78–3.98 g GAE/100 g. Subcritical water extraction in an inert nitrogen atmosphere generally resulted in extracts with the higher antioxidant activity, with hemp and flax oilseed cakes exhibiting the highest antioxidant activities (15.0 ± 1.2 to 25.1 ± 0.8 mg TE/g dw). In the ABTS assay, the values ranged from 18.6 ± 1.3 to 40.6 ± 1.9 mg AAE/g dw. Antioxidant activities were in agreement with the content of phenolic and flavonoid compounds.

A total of 29 different polyphenolic compounds were identified in the extracts. The flavonol myricetin was detected in all analysed samples for all tested conditions with values ranging from 28.1 ± 1.4 to 57.2 ± 2.9 mg/100 dw. ( +)-Catechin was also quantified in all samples, with concentrations at least threefold higher in hemp extracts than in the pumpkin or flax oilseed cakes. In the case of (−)-epicatechin, the highest level was found in flax (27.9 ± 1.4 to 33.2 ± 1.6 mg/100 dw), followed by hemp (10.1 ± 0.4 to 18.8 ± 0.9 mg/100 dw) and pumpkin (3.93 ± 0.20 to 11.8 ± 0.6 mg/100 dw). In the case of compounds from the phenolic acid family, gallic acid was one of the major contributors to the total amount of phenolic compounds quantified in oilseed cakes, with the highest values reported for the samples extracted using the acidic modifier.

Cellulose hydrolysis with the acid catalyst led to the highest glucose concentrations, being several folds higher in comparison to other treatment conditions for all oilseed cakes. The highest in glucose was hemp hydrolysate (255.54 mg/l). Xylose, originating from hemicellulose fraction, in moderate concentrations was determined in flax and hemp seed samples, whereas in pumpkin seed cake hydrolysates it was very low. Arabinose originating from hemicellulose as well, was the highest in the flax cake hydrolysates obtained in carbon-dioxide atmosphere (336.33 mg/l). The formation of acetic acid was pronounced in all samples, contributing to the acidity of the hydrolysates. The highest content, however, was in the pumpkin seed hydrolysate (743.67 mg/l).

The results of sugar degradation products were in agreement with the glucose content. The highest contents of 5-HMF and furfural were determined in flax hydrolysates, being 112.44 mg/l and 137.90 mg/l, respectively.

The hemp and pumpkin seed hydrolysates showed a considerable degree of cytotoxicity in Caco-2 cells (viability of 8.93 ± 1.17% and 61.93 ± 9.77%, respectively). In HT29-MTX cells all oilseed hydrolysates exhibited similar results, i.e. the viabilities observed for all tested concentrations were always above 75%, not classifying them as cytotoxic.

## Data Availability

The datasets used in this study are available from the corresponding author on reasonable request.

## Abbreviations

SWE: Subcritical water extraction
TPC: Total phenol content
TFC: Total flavonoid content
FRAP: Ferric reducing ability of plasma
DPPH: 2,2-Diphenyl-1-picrylhydrazyl
HPLC: High-pressure liquid chromatography
ABTS: 2,2′-Azino-bis(3-ethylbenzothiazoline-6-sulfonic acid
5-MF: 5-Methyl-furfural
5-HMF: 5-Hydroxymethylfurfural
TPTZ: 2,4,6-Tris(2-pyridyl)-s-triazine
AA: Ascorbic acid
DMSO: Dimethylsulfoxide
